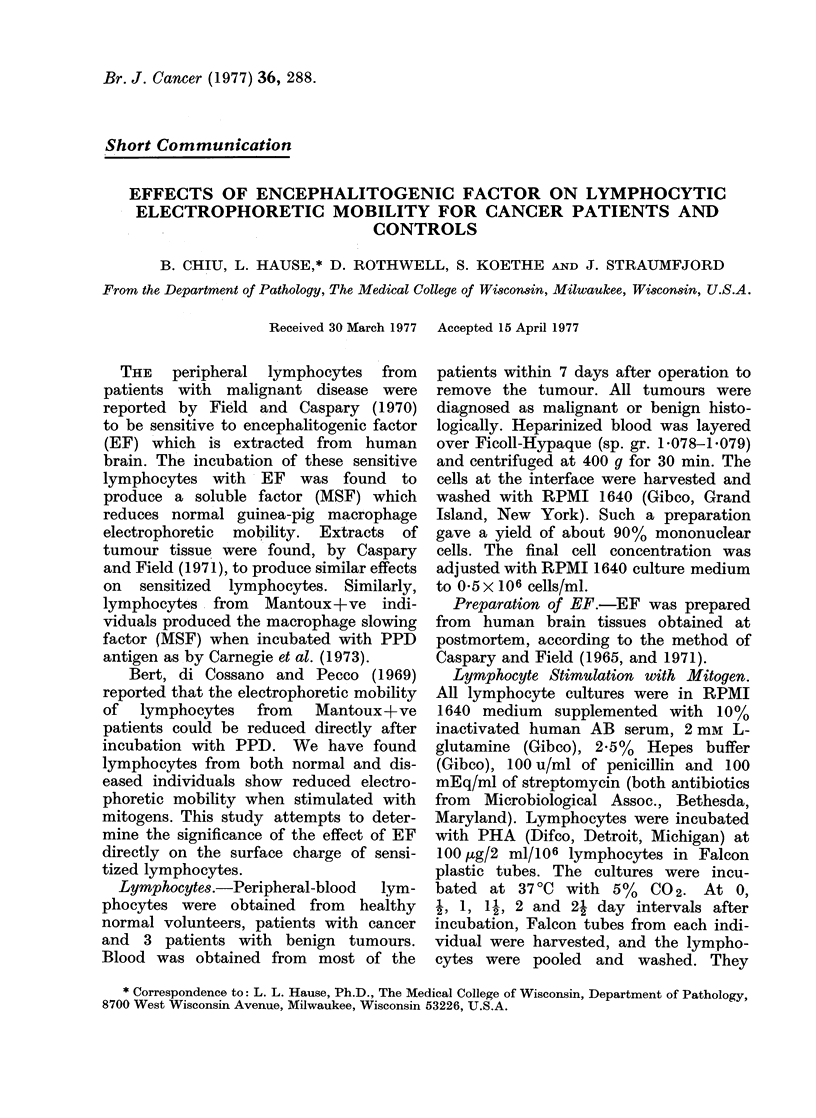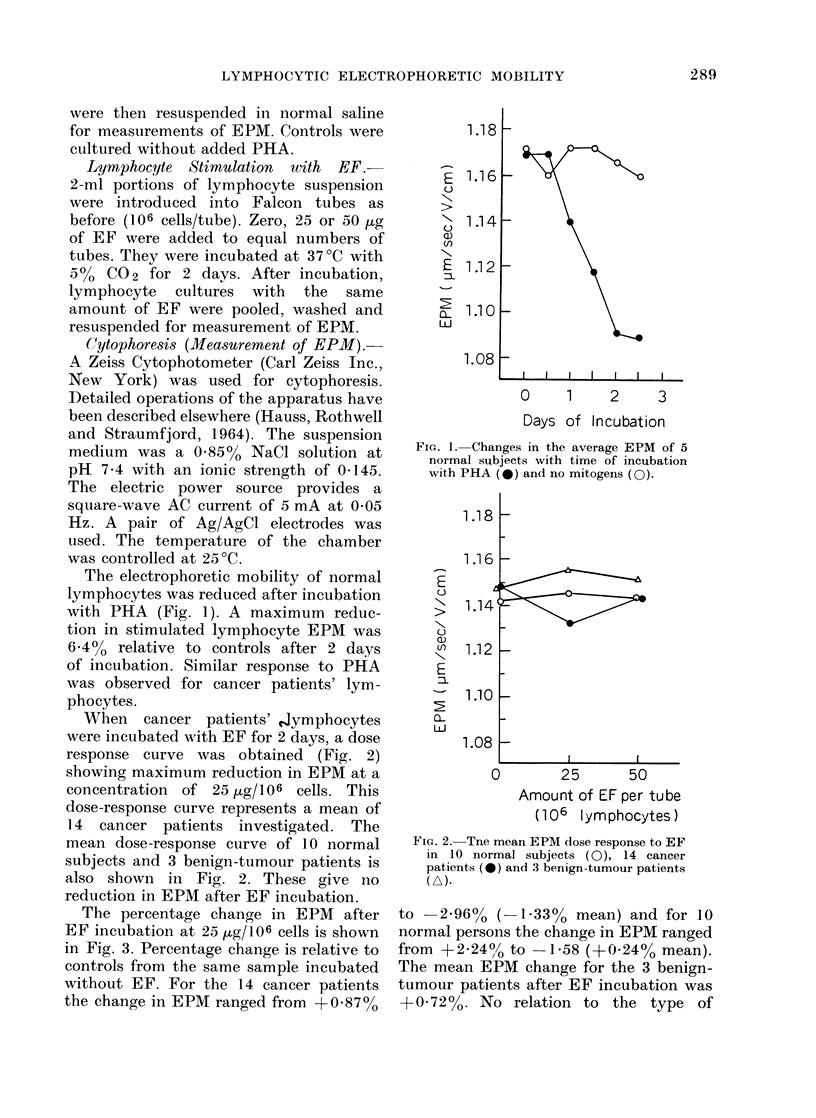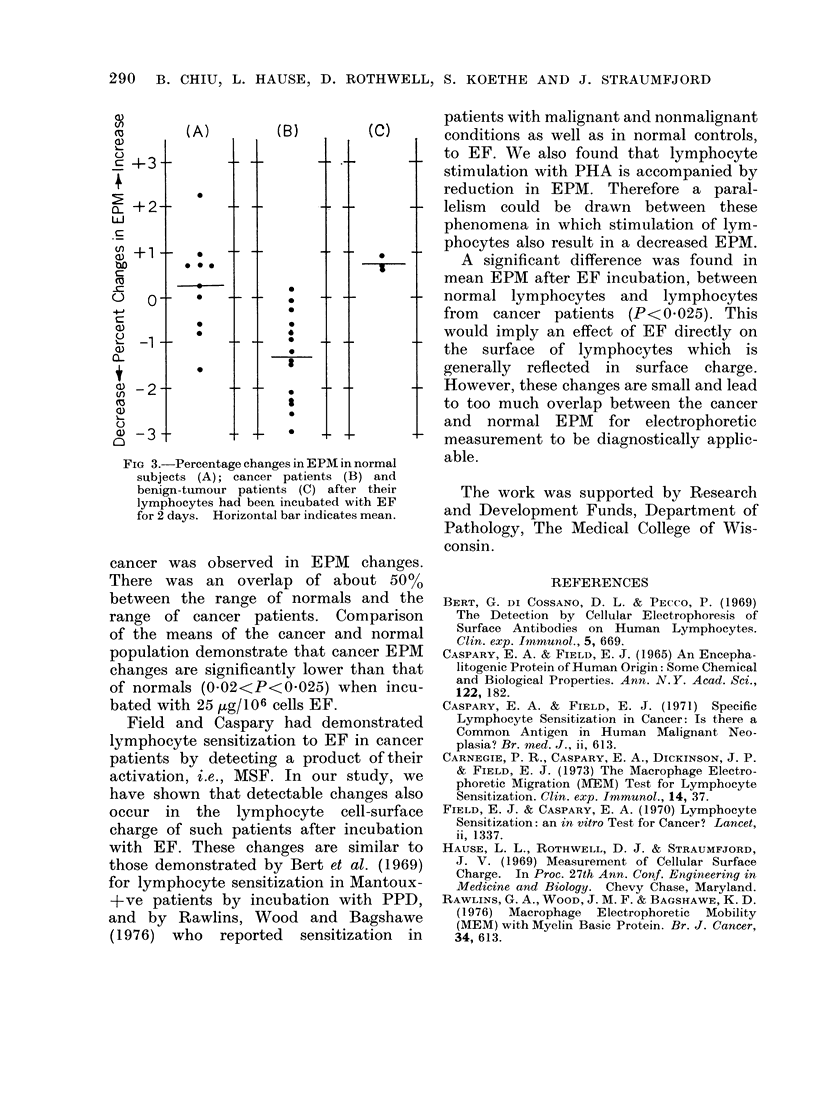# Effects of encephalitogenic factor on lymphocytic electrophoretic mobility for cancer patients and controls.

**DOI:** 10.1038/bjc.1977.189

**Published:** 1977-08

**Authors:** B. Chiu, L. Hause, D. Rothwell, S. Koethe, J. Straumfjord


*Br. J. Cancer* (1977) **36**, 288.

***Short Communication***

### EFFECTS OF ENCEPHALITOGENIC FACTOR ON LYMPHOCYTIC ELECTROPHORETIC MOBILITY FOR CANCER PATIENTS AND CONTROLS

B. CHIU, L. HAUSE,* D. ROTHWELL, S. KOETHE AND J. STRAUMFJORD

*From the Department of Pathology, The Medical College of Wisconsin, Milwaukee, Wisconsin, U.S.A.*

Received 30 March 1977 Accepted 15 April 1977

THE peripheral lymphocytes from patients with malignant disease were reported by Field and Caspary (1970) to be sensitive to encephalitogenic factor (EF) which is extracted from human brain. The incubation of these sensitive lymphocytes with EF was found to produce a soluble factor (MSF) which reduces normal guinea-pig macrophage electrophoretic mobility. Extracts of tumour tissue were found, by Caspary and Field (1971), to produce similar effects on sensitized lymphocytes. Similarly, lymphocytes from Mantoux+ve individuals produced the macrophage slowing factor (MSF) when incubated with PPD antigen as by Carnegie *et al.* (1973).

Bert, di Cossano and Pecco (1969) reported that the electrophoretic mobility of lymphocytes from Mantoux+ve patients could be reduced directly after incubation with PPD. We have found lymphocytes from both normal and diseased individuals show reduced electrophoretic mobility when stimulated with mitogens. This study attempts to determine the significance of the effect of EF directly on the surface charge of sensitized lymphocytes.

*Lymphocytes.*—Peripheral-blood lymphocytes were obtained from healthy normal volunteers, patients with cancer and 3 patients with benign tumours. Blood was obtained from most of the patients within 7 days after operation to remove the tumour. All tumours were diagnosed as malignant or benign histologically. Heparinized blood was layered over Ficoll-Hypaque (sp. gr. 1·078–1·079) and centrifuged at 400 *g* for 30 min. The cells at the interface were harvested and washed with RPMI 1640 (Gibco, Grand Island, New York). Such a preparation gave a yield of about 90% mononuclear cells. The final cell concentration was adjusted with RPMI 1640 culture medium to $0{\cdot}5 \times 10^6$ cells/ml.

*Preparation of EF.*—EF was prepared from human brain tissues obtained at postmortem, according to the method of Caspary and Field (1965, and 1971).

*Lymphocyte Stimulation with Mitogen.* All lymphocyte cultures were in RPMI 1640 medium supplemented with 10% inactivated human AB serum, 2 mM L-glutamine (Gibco), 2·5% Hepes buffer (Gibco), 100 u/ml of penicillin and 100 mEq/ml of streptomycin (both antibiotics from Microbiological Assoc., Bethesda, Maryland). Lymphocytes were incubated with PHA (Difco, Detroit, Michigan) at 100 $\mu$g/2 ml/$10^6$ lymphocytes in Falcon plastic tubes. The cultures were incubated at 37 °C with 5% $CO_2$. At 0, $\frac{1}{2}$, 1, $1\frac{1}{2}$, 2 and $2\frac{1}{2}$ day intervals after incubation, Falcon tubes from each individual were harvested, and the lymphocytes were pooled and washed. They

* Correspondence to: L. L. Hause, Ph.D., The Medical College of Wisconsin, Department of Pathology, 8700 West Wisconsin Avenue, Milwaukee, Wisconsin 53226, U.S.A.

were then resuspended in normal saline for measurements of EPM. Controls were cultured without added PHA.

*Lymphocyte Stimulation with EF*.—2-ml portions of lymphocyte suspension were introduced into Falcon tubes as before ($10^6$ cells/tube). Zero, 25 or 50 μg of EF were added to equal numbers of tubes. They were incubated at 37 °C with 5% $CO_2$ for 2 days. After incubation, lymphocyte cultures with the same amount of EF were pooled, washed and resuspended for measurement of EPM.

*Cytophoresis (Measurement of EPM)*.—A Zeiss Cytophotometer (Carl Zeiss Inc., New York) was used for cytophoresis. Detailed operations of the apparatus have been described elsewhere (Hauss, Rothwell and Straumfjord, 1964). The suspension medium was a 0·85% NaCl solution at pH 7·4 with an ionic strength of 0·145. The electric power source provides a square-wave AC current of 5 mA at 0·05 Hz. A pair of Ag/AgCl electrodes was used. The temperature of the chamber was controlled at 25 °C.

The electrophoretic mobility of normal lymphocytes was reduced after incubation with PHA (Fig. 1). A maximum reduction in stimulated lymphocyte EPM was 6·4% relative to controls after 2 days of incubation. Similar response to PHA was observed for cancer patients' lymphocytes.

When cancer patients' lymphocytes were incubated with EF for 2 days, a dose response curve was obtained (Fig. 2) showing maximum reduction in EPM at a concentration of 25 μg/$10^6$ cells. This dose-response curve represents a mean of 14 cancer patients investigated. The mean dose-response curve of 10 normal subjects and 3 benign-tumour patients is also shown in Fig. 2. These give no reduction in EPM after EF incubation.

FIG. 1.—Changes in the average EPM of 5 normal subjects with time of incubation with PHA (●) and no mitogens (○).

FIG. 2.—Tne mean EPM dose response to EF in 10 normal subjects (○), 14 cancer patients (●) and 3 benign-tumour patients (△).

The percentage change in EPM after EF incubation at 25 μg/$10^6$ cells is shown in Fig. 3. Percentage change is relative to controls from the same sample incubated without EF. For the 14 cancer patients the change in EPM ranged from +0·87% to −2·96% (−1·33% mean) and for 10 normal persons the change in EPM ranged from +2·24% to −1·58 (+0·24% mean). The mean EPM change for the 3 benign-tumour patients after EF incubation was +0·72%. No relation to the type of

FIG 3.—Percentage changes in EPM in normal subjects (A); cancer patients (B) and benign-tumour patients (C) after their lymphocytes had been incubated with EF for 2 days. Horizontal bar indicates mean.

cancer was observed in EPM changes. There was an overlap of about 50% between the range of normals and the range of cancer patients. Comparison of the means of the cancer and normal population demonstrate that cancer EPM changes are significantly lower than that of normals ($0{\cdot}02 < P < 0{\cdot}025$) when incubated with 25 $\mu$g/$10^6$ cells EF.

Field and Caspary had demonstrated lymphocyte sensitization to EF in cancer patients by detecting a product of their activation, *i.e.*, MSF. In our study, we have shown that detectable changes also occur in the lymphocyte cell-surface charge of such patients after incubation with EF. These changes are similar to those demonstrated by Bert *et al.* (1969) for lymphocyte sensitization in Mantoux-+ve patients by incubation with PPD, and by Rawlins, Wood and Bagshawe (1976) who reported sensitization in patients with malignant and nonmalignant conditions as well as in normal controls, to EF. We also found that lymphocyte stimulation with PHA is accompanied by reduction in EPM. Therefore a parallelism could be drawn between these phenomena in which stimulation of lymphocytes also result in a decreased EPM.

A significant difference was found in mean EPM after EF incubation, between normal lymphocytes and lymphocytes from cancer patients ($P < 0{\cdot}025$). This would imply an effect of EF directly on the surface of lymphocytes which is generally reflected in surface charge. However, these changes are small and lead to too much overlap between the cancer and normal EPM for electrophoretic measurement to be diagnostically applicable.

The work was supported by Research and Development Funds, Department of Pathology, The Medical College of Wisconsin.

### REFERENCES

BERT, G. DI COSSANO, D. L. & PECCO, P. (1969) The Detection by Cellular Electrophoresis of Surface Antibodies on Human Lymphocytes. *Clin. exp. Immunol.*, **5**, 669.

CASPARY, E. A. & FIELD, E. J. (1965) An Encephalitogenic Protein of Human Origin: Some Chemical and Biological Properties. *Ann. N.Y. Acad. Sci.*, **122**, 182.

CASPARY, E. A. & FIELD, E. J. (1971) Specific Lymphocyte Sensitization in Cancer: Is there a Common Antigen in Human Malignant Neoplasia? *Br. med. J.*, ii, 613.

CARNEGIE, P. R., CASPARY, E. A., DICKINSON, J. P. & FIELD, E. J. (1973) The Macrophage Electrophoretic Migration (MEM) Test for Lymphocyte Sensitization. *Clin. exp. Immunol.*, **14**, 37.

FIELD, E. J. & CASPARY, E. A. (1970) Lymphocyte Sensitization: an *in vitro* Test for Cancer? *Lancet*, ii, 1337.

HAUSE, L. L., ROTHWELL, D. J. & STRAUMFJORD, J. V. (1969) Measurement of Cellular Surface Charge. In *Proc. 27th Ann. Conf. Engineering in Medicine and Biology.* Chevy Chase, Maryland.

RAWLINS, G. A., WOOD, J. M. F. & BAGSHAWE, K. D. (1976) Macrophage Electrophoretic Mobility (MEM) with Myelin Basic Protein. *Br. J. Cancer*, **34**, 613.